# Identification of errors introduced during high throughput sequencing of the T cell receptor repertoire

**DOI:** 10.1186/1471-2164-12-106

**Published:** 2011-02-11

**Authors:** Phuong Nguyen, Jing Ma, Deqing Pei, Caroline Obert, Cheng Cheng, Terrence L Geiger

**Affiliations:** 1 Department of Pathology, St. Jude Children's Research Hospital, 262 Danny Thomas Pl., Memphis, TN 38105, USA; 2 Department of Information Sciences, St. Jude Children's Research Hospital, 262 Danny Thomas Pl., Memphis, TN 38105, USA; 3 Department of Biostatistics, St. Jude Children's Research Hospital, 262 Danny Thomas Pl., Memphis, TN 38105, USA; 4 Hartwell Center for Bioinformatics, St. Jude Children's Research Hospital, 262 Danny Thomas Pl., Memphis, TN 38105, USA

## Abstract

**Background:**

Recent advances in massively parallel sequencing have increased the depth at which T cell receptor (TCR) repertoires can be probed by >3log10, allowing for saturation sequencing of immune repertoires. The resolution of this sequencing is dependent on its accuracy, and direct assessments of the errors formed during high throughput repertoire analyses are limited.

**Results:**

We analyzed 3 monoclonal TCR from TCR transgenic, Rag^-/- ^mice using Illumina^® ^sequencing. A total of 27 sequencing reactions were performed for each TCR using a trifurcating design in which samples were divided into 3 at significant processing junctures. More than 20 million complementarity determining region (CDR) 3 sequences were analyzed. Filtering for lower quality sequences diminished but did not eliminate sequence errors, which occurred within 1-6% of sequences. Erroneous sequences were pre-dominantly of correct length and contained single nucleotide substitutions. Rates of specific substitutions varied dramatically in a position-dependent manner. Four substitutions, all purine-pyrimidine transversions, predominated. Solid phase amplification and sequencing rather than liquid sample amplification and preparation appeared to be the primary sources of error. Analysis of polyclonal repertoires demonstrated the impact of error accumulation on data parameters.

**Conclusions:**

Caution is needed in interpreting repertoire data due to potential contamination with mis-sequence reads. However, a high association of errors with phred score, high relatedness of erroneous sequences with the parental sequence, dominance of specific nt substitutions, and skewed ratio of forward to reverse reads among erroneous sequences indicate approaches to filter erroneous sequences from repertoire data sets.

## Background

It is estimated that >2 × 10^6 ^and 2 × 10^7 ^unique T cell clones reside in the lymphoid organs and circulation of mice and humans respectively [1,2]. Each of these expresses a single or occasionally two unique TCRs that provide for antigen specificity. Somatic recombination and associated N and P region mutagenesis of TCR gene segments in developing thymocytes allows for the possible formation of >10^15 ^unique TCRs. Receptor diversity is focused on a small segment of the TCRα and β chain genes, the CDR3 [3]. This ~8-14 amino acid long segment most directly engages antigenic peptides bound to restricting major histocompatibility complex (MHC) molecules and provides much of the specificity in TCR recognition.

On average, 20 - 200 T cells of a single clone are estimated to reside in the circulation [4]. However, clonal frequencies may vary dramatically depending on a cell's specificity and immunologic history. In the setting of infection and immunity oligoclonal expansions may comprise a substantial proportion of the T cell population [5,6]. Analyses of the TCR repertoire have provided insight into the nature and dynamics of these immune responses [7-11]. A variety of approaches have been used, however ultimately direct sequence analyses of CDR3 provide the most detailed information about the T cell clones present.

In the past, sequence analysis of TCR repertoires was laborious, requiring the cloning of individual TCR cDNA. Typically dozens to hundreds of sequences were obtained [12,13]. At the upper end, such studies could robustly identify sequences more common than several percent of the repertoire, but only sparsely sample the majority of TCR clones that are of lower frequency. More recently, high throughput, or massively parallel, sequencing technologies have allowed the rapid and simultaneous acquisition of up to millions of independent sequences [14-18]. This has the potential to interrogate TCR sequences over a much larger frequency range.

High throughput approaches are most commonly used for sequencing bulk genomic DNA or cDNA, and oversequencing facilitates the identification and correction of randomly introduced errors [19,20]. In repertoire analysis, excluding erroneous sequences is less straightforward. The developmental introduction of nucleotide (nt) additions, deletions, and substitutions in the CDR3 allows for the generation of large numbers of sequences that do not conform to a genomic template. Further, despite the enormous potential for diversity, CDR3 sequences show biases [21,22]. The frequency of a specific TCR may vary tremendously within a population and selection pressure, either developmental or during an immune response, may promote the survival and expansion of specific CDR3, sometimes with convergent but non-identical sequences [12,23]. Therefore, highly variable CDR3 frequencies and subtle sequence differences among CDR3 may exist and do not necessarily indicate mis-sequencing events.

Two platforms have been used for massively parallel sequencing of immune repertoires, those from Roche and Illumina^® ^[19]. With each, sequence reads sufficiently long to encompass a CDR3 are feasible. Here we use monoclonal TCR to assess the frequency, types, and sources of errors introduced into TCR using Illumina^® ^sequencing. Our findings indicate a considerable error frequency of ~1-6%. They also reveal sequence features that may be used to purge erroneous sequences, and thereby enhance confidence in repertoire data.

## Results

### Experimental design

To estimate the frequency and isolate the sources of erroneous sequences acquired during TCR repertoire analysis, we analyzed cells from Rag^-/- ^mice transgenic for either of 3 rearranged TCRαβ, 5C.C7, OT-1, and DO11.10. Because of the Rag deficiency, these cells only express the rearranged transgenic TCR, allowing absolute identification of errors accruing during sample preparation or through mis-sequencing. Splenic cDNA was split into 3 samples from which CDR3β amplification was performed using Cβ and Vβ-specific primers. The amplicons were split again into 3, sequencing primers ligated, and second stage amplification performed. The products were further divided into 3, and single-end 125 bp reads acquired using the Illumina^® ^Genome Analyzer IIx in separate lanes. Using this trifurcating design, 27 independent sequencing reactions were performed for each of the 3 monoclonal TCR. CDR3β nt sequences were analyzed beginning from the codon following the conserved 5' C to that immediately preceding the conserved 3' F. This sequence was 3 (5C.C7, OT-1) or 7 nt (DO11.10) distal to the V region primer and 33 nt from the Cβ primer. CDR3β length was 36 nt (5C.C7, DO11.10) or 30 nt (OT-1). Sequences that were out of frame, contained unassigned "N" nucleotides, or lacked complete identity with conserved Vβ and Jβ regions external to the CDR3 were discarded. A total of 9,107,256 5C.C7, 5,004,141 OT-1, and 6,123,180 DO11.10 CDR3β sequences were obtained.

### Similar total error rates for distinct TCR

**Figure 1:**
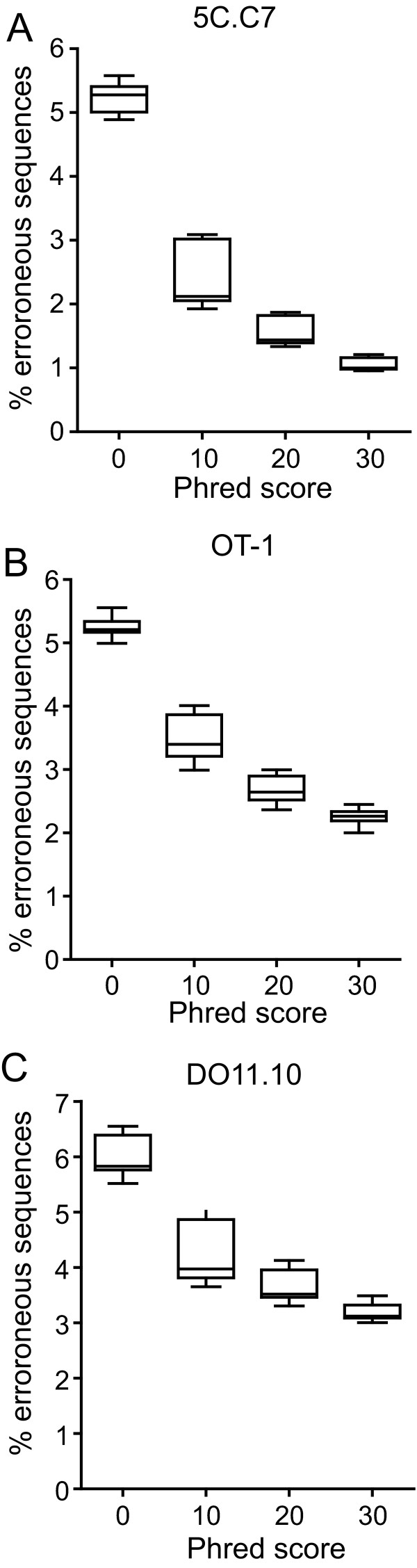
**Error rate of sequencing reactions**. Box and whisker plots show median, 25-75 percentile, and range for erroneous sequences for the 5C.C7 (A), OT-1 (B), and DO11.10 (C) CDR3 sequences expressed as a percent of total sequence events that met initial criteria. Phred values were used to further constrain sequence sets and the minimal phred cutoff score for any nt in a sequence is indicated.

The overall rate of erroneous CDR3β sequences for the 3 TCR was similar, 5.23 ± 0.21%, 5.24 ± 0.12%, and 6.00 ± 0.34% for the 5C.C7, OT-1, and DO11.10 TCR respectively. We analyzed whether additional filtering of sequence based on quality scores could selectively reduce the percent of erroneous sequences. Increasing minimal nt phred (q) values from 0 - 30 led to a progressive reduction in errors (Figure 1a-c)[24]. At a q = 30, net error rate was reduced to 1.05 ± 0.12%, 2.25 ± 0.12%, and 3.19 ± 0.14% for the 5C.C7, OT-1, and DO11.10 sequences, a 47-80% reduction in the different TCR (Figure 1a-c). This was accompanied by a reduction in the total number of evaluable sequences by between 24.3 ± 3.6% (5C.C7) to 35.3 ± 4.3% (OT-1). However, the exclusion of erroneous sequences exceeded that of correct sequences, compensating for this loss in total sequence numbers (see Additional file 1, Supp. Figure S1a, b).

Importantly, there was a dramatic decrease in the total number of unique errant sequences acquired with increasing phred cutoffs. Individual data sets showed mean reductions ranging from 86% (OT-1 TCR) to 94% (5C.C7 TCR) (see Additional file 1, Supp. Figure S1c). Pooled data sets generated by combining results from the 27 samples sequenced for each TCR showed a more dramatic reduction of between 94% (DO11.10 TCR, reduction from 14,280 unique errant sequences to 911) and 98% (5C.C7 TCR, reduction from 31,757 to 532) (see Additional file 1, Supp. Figure S1d). Therefore, high frequency parental sequences may be associated with large numbers of derivative erroneous sequences. Further, with increasing data set size, phred cutoffs limit erroneous sequences to a smaller sequence pool.

### Quantity and composition of erroneous sequences

**Figure 2:**
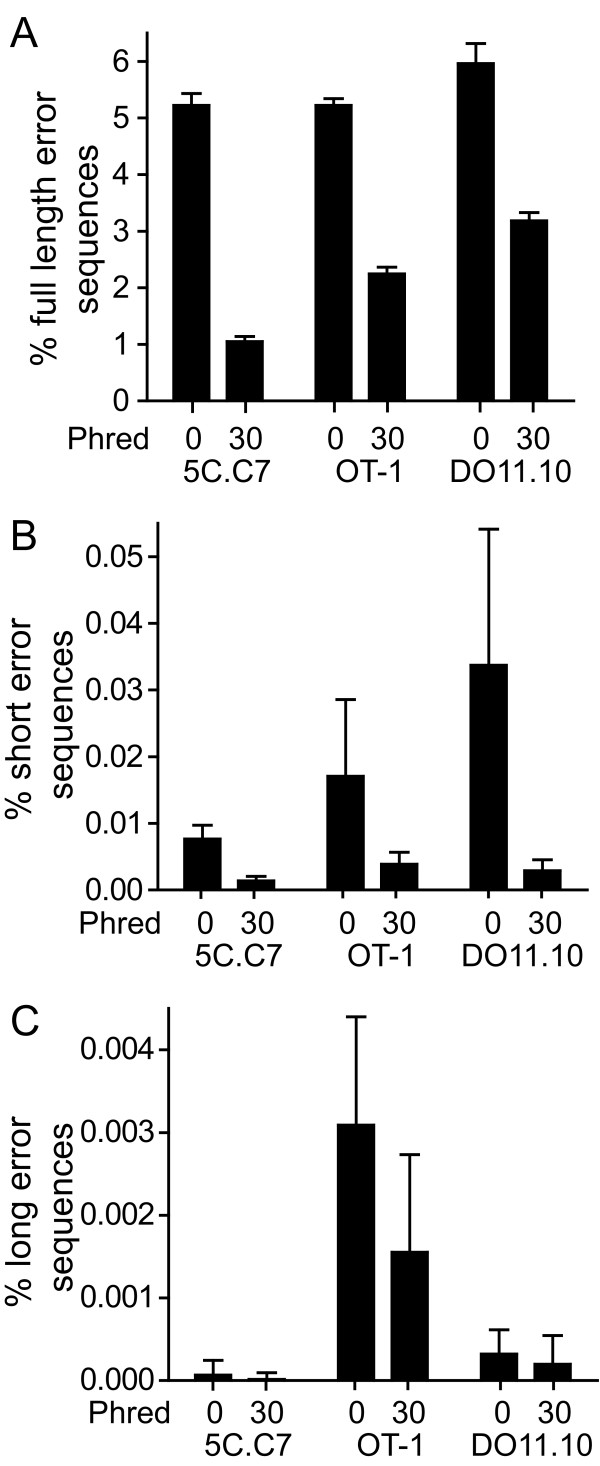
**Length of erroneous sequences**. Mean+1 S.D. of the percent of total sequences of errors of same length (A), shortened (B), and elongated (C) compared with the correct sequence is plotted. Sequences filtered for a minimal phred score of 30 for each nt is compared with sequences not filtered for phred value.

**Figure 3:**
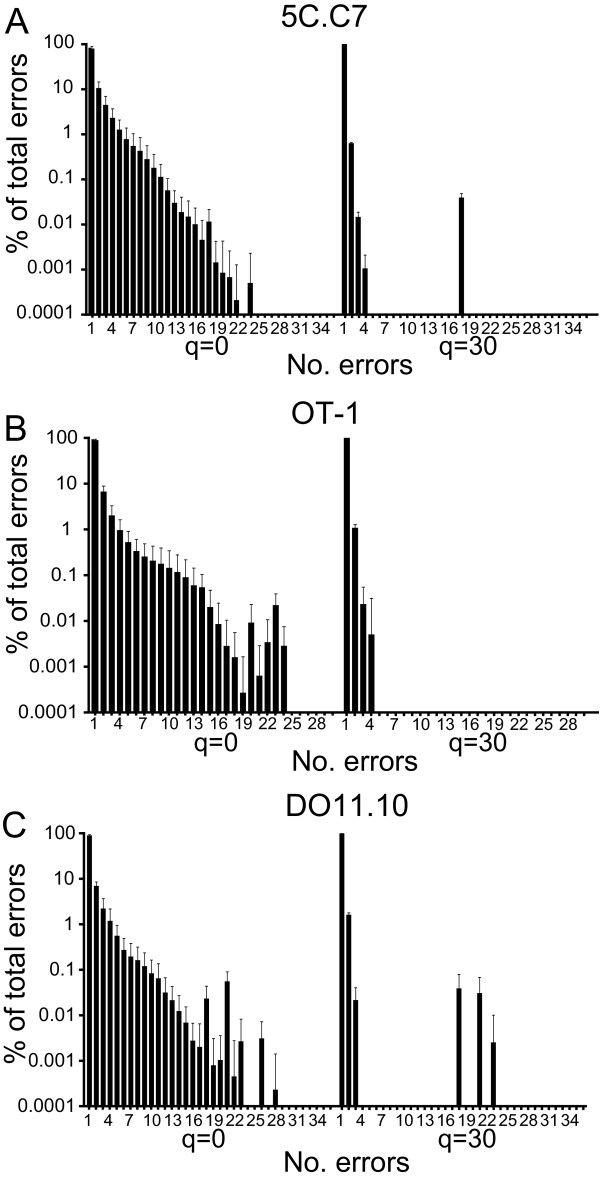
**Multiple errors in CDR3 sequences**. Plot shows mean+1 s.d. of the frequency of correct-length erroneous sequences with the indicated number of nt substitutions as a percent of the total correct-length erroneous sequences for the 5C.C7 (A), OT-1 (B), and DO11.10 TCR, and with phred cutoff scores of 0 or 30.

Virtually all of the erroneous sequences were of the correct length, and this comprised >99% of the error population acquired either with a q = 0 or 30. Most of the remaining sequences were truncated (Figure 2a-c). Among erroneous sequences of correct length that were not filtered based on phred score, an average of 79%, 88%, and 88% for 5C.C7, OT-1, and DO11.10 had a single error, with progressively decreasing frequency of sequences with greater numbers of errors (Figure 3a-c). Increasing the q value to 30 increased the percent of erroneous sequences with single nt replacements to >98% for each of the TCR.

**Figure 4:**
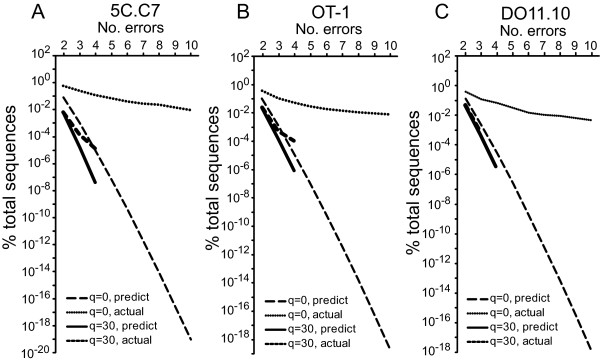
**Complementation in error occurrence**. An expected frequency of multiple errors was calculated based on the assumption that each error is independent using the formula p = C(SER)^M^, where SER = observed single error rate, M = number of mutated nt in sequence, and C = total number of possible erroneous sequence combinations. C = N!/(M!x(N-M)!), where N = number of nucleotides in the sequence. The expected frequency of multiple mutations is plotted against the observed frequency in experimental samples either for data sets not filtered based on phred score or filtered at a q = 30, and for the presence of between 2 and 10 mutated nt for q = 0 and 2 and 4 for q = 30 (no events were observed with 4-10 mutations for q = 30 filtered data).

Although even with a q = 0 only ~1% of total sequences had multiple errors, their incidence was greater than that which would have been anticipated from the single error rate. This indicates complementation in the formation of multiple errors (Figure 4a-c). Screening using a q = 30, however, reduced the number of sequences with multiple errors, and multiple error rates more closely reflected those predicted from the single error rate. Therefore, a substantial proportion of sequences, ~1 - 6% depending on the TCR and use of phred quality filtering, acquired using high throughput sequencing were erroneous, these sequences were virtually exclusively of the correct length, and primarily single nt substitutions. Filtering sequences based on phred scores altered both the quantity and the types of errors observed.

**Figure 5:**
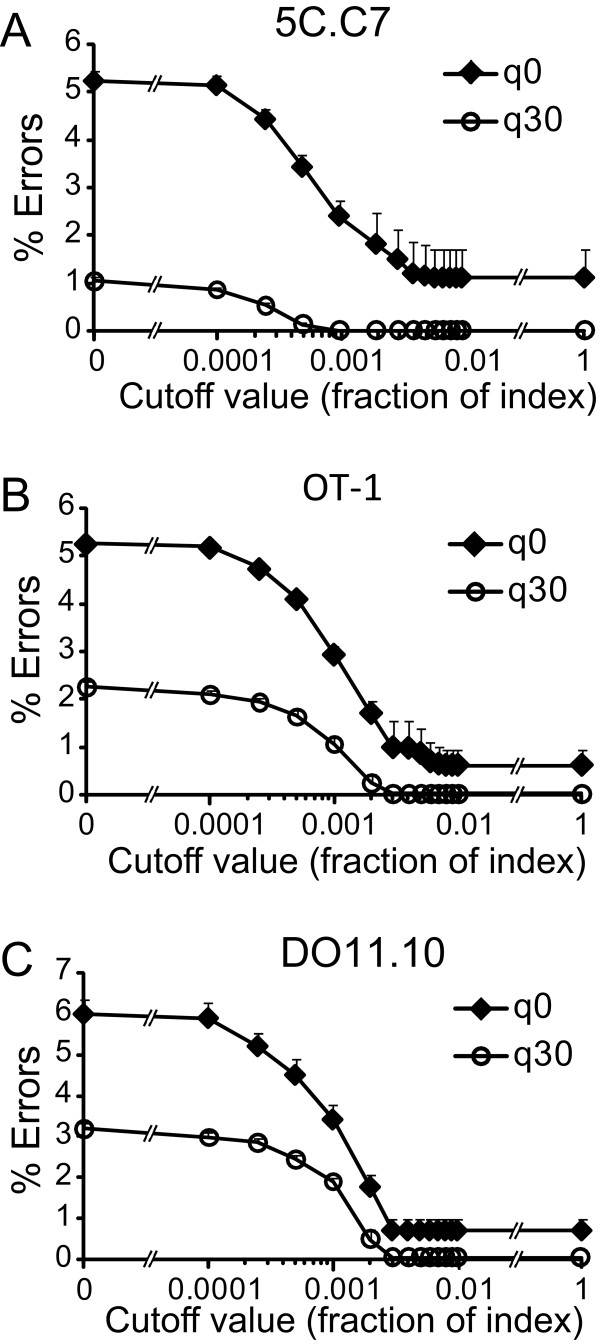
**Filtering single nt mismatch sequences from repertoire data**. To determine the extent to which errors could be purged by filtering sequences with single nt mismatches, we examined the residual percent of erroneous sequences for each sequencing reaction after culling single nt mismatch sequences. Assessment of residual erroneous sequences was performed at multiple cutoff values for the frequency of the mismatch sequence relative to the true 5C.C7 (A), OT-1 (B), or DO11.10 (C) sequence, and mean + 1 s.d. plotted. Our data suggests values of less than 0.01 are adequate for optimal error reduction. In application, a cutoff would need to be selected that optimizes removal of erroneous sequences while also minimizing inadvertent culling of true sequences.

Considering that a majority of erroneous sequences are single nt substitutions, it should be possible to purge erroneous sequences by excluding sequences present at lower frequency and differing by a single nt from an index sequence. To assess this, we calculated the impact of filtering sequences with single nt mismatches compared with the true 5C.C7, OT-1, and DO11.10 TCR CDR3. Cutoff values, indicating the maximum frequency of the culled single nt mismatch sequence relative to the correct CDR3 sequence, were varied (Figure 5a-c). An exclusion cutoff of 0 does not filter any of the sequences. A cutoff of 1 eliminates all single nt mismatches at or below the index sequence's frequency. Residual erroneous sequences at this latter cutoff include multiple sequence mismatches, or nt additions or deletions. For each TCR, cutoffs in the range of 0.0001-0.01 (0.01-1% of index frequency) eliminated most erroneous sequences. Indeed, at a q = 30 and cutoff of 0.01, only 0.0086 ± 0.002%, 0.030 ± 0.006%, and 0.057 ± 0.008% of total sequences were erroneous for the 5C.C7, OT-1, and DO11.10 CDR3 respectively. Therefore filtering single nt mismatch sequences has the potential to dramatically decrease overall error rates in CDR3 acquired by next generation sequencing.

### Asymmetric incorporation of errors

To better define the errors incorporated during sequencing, we analyzed both their positional dependence and the sources of variation in their frequency. Errors were tabulated by nt position and substitution. Rates of errors varied substantially with different sites and nt substitutions. For the OT-1 TCR, the least common error was a 30C→A substitution, identified on average in 0.0016% of sequences, whereas the most common error, 17A→C, was observed in 0.55%, a ~350-fold difference. Similarly divergent site and nt-dependent error rates were observed in the 5C.C7 and DO11.10 sequencing, with 44 and 46-fold differences observed between the least and most common substitutions.

**Figure 6:**
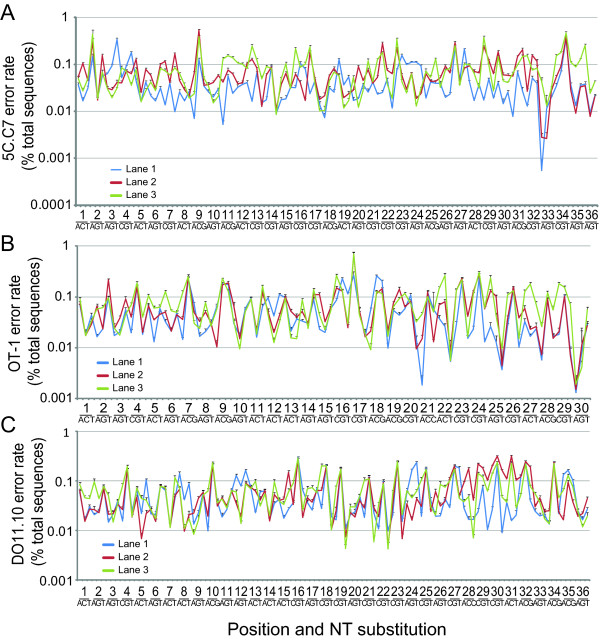
**Position and nt specific substitutions**. The frequency of sequences with the indicated nt substitutions among total acquired, phred unfiltered sequences is plotted for 5C.C7 (A), OT-1 (B), and DO11.10 TCR (C). Mean+1 s.d. of 9 samples per lane is plotted for each of the 3 sequencing lanes to highlight lane-specific differences in position/nt error rates. Plotted lines are shown to aid visualization of results from single lanes and do not indicate continuity among x-axis variables.

Within this variability, the sequencing lane significantly influenced error frequency for individual samples (Figure 6a-c). As examples, in the DO11.10 sequencing, lane 1 showed an anomalously high error rate for 8G→T and 24C→T substitutions, whereas lane 2 showed a particularly low rate of 5G→C errors. Rates of many other errors, such as 7G→C and 16A→G, were nearly identical across lanes. In contrast to the prominent lane effect, error rates showed little correspondence with the preparatory pathway taken. Concordance in error rates among the 9 differentially prepared samples of each TCR loaded per lane was reflected in their low standard deviations. Indeed, the composite co-efficient of variation (CV) for the individual lane-segregated values in Figure 6a-c were 0.10 ± 0.04 for the 5C.C7 TCR, 0.15 ± 0.20 for OT-1, and 0.12 ± 0.06 for DO11.10. This contrasted with the several fold larger CVs obtained when site specific error rates were analyzed in a lane-independent manner, where values of 0.49 ± 0.24, 0.44 ± 0.27, and 0.39 ± 0.25 respectively were obtained (not shown).

Variability in rates of specific site and nt-substitutions remained after application of a q = 30 filter. However, the lane-dependence on error rates was markedly diminished and this was reflected in the low s.d. observed when sample data was averaged across lanes (see Additional file 2, Supp. Figure S2a-c). Indeed, the number of site-specific nt substitutions significant across lanes by ANOVA (p < 0.05) decreased from 95% to 27%, 84% to 11%, and 82% to 13% of all possible errors for the 5C.C7, OT-1, and DO11.10 TCR respectively by increasing the phred cutoff from 0 to 30. Although the overall location and nt substitution-specific error rate was lower for the phred-filtered sequences than for unfiltered sequences at all positions, substantial variability was nevertheless observed. A frequency of >0.1% was observed for some locations/nt substitutions, whereas for others no errors were found among the millions of sequences acquired. Therefore, errors show significant site and nt-specific variability regardless of the application of a phred filter for sequence quality. The low s.d. observed for independently prepared samples indicates that idiosyncratic introduction of errors during sample preparation does not markedly influence the rate, type, and locations of sequence errors.

### Sequence direction influence on error frequency

Templates may bind to the Illumina^® ^solid phase in either a 5'→3' or 3'→5' orientation, where they undergo a solid phase amplification followed by a unidirectional sequencing reaction. Errors may be dependent on the orientation of the strand, with distinct rates for reads from the complementary sequences. To assess for this, we explored the presence of errors in templates read either in a "forward" or "reverse" orientation.

**Figure 7:**
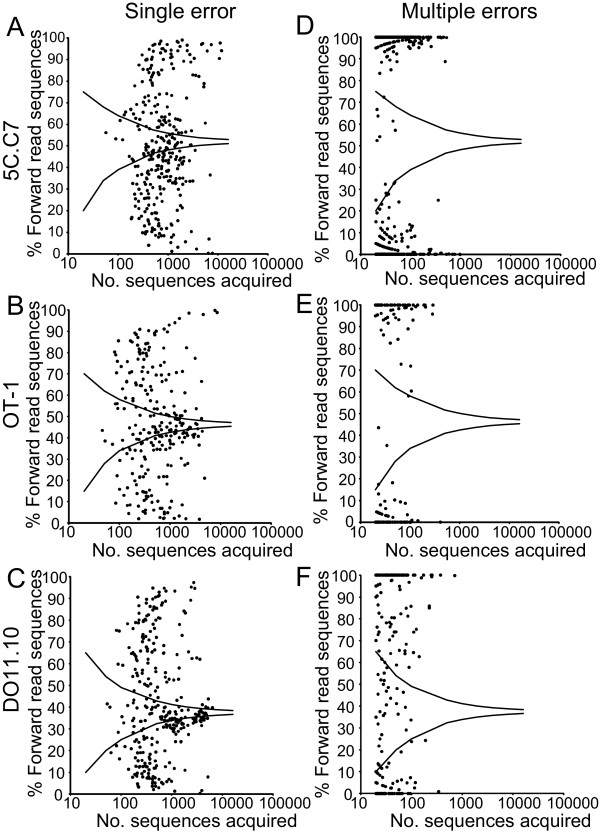
**Skewing in sequence read direction**. The number of reads performed in a forward or reverse orientation were tabulated for each phred unfiltered, erroneous sequence for which a total of >20 independent sequences were acquired in a lane. Percent forward reads is plotted on the ordinate versus number of sequences acquired in the abscissa. If read direction during sequencing was random, data points would be anticipated to fall within a binomial distribution centered on the value obtained for correct sequence reads. Plotted curves indicate calculated boundaries of the upper and lower limits of values between which 98% of sequences should be found. These were calculated using the Vassar binomial calculator http://faculty.vassar.edu/lowry/binomialX.html with p = probability of forward read among correct sequences, n = number of reads (abscissa), and defining the number of positive events for which the probability of identifying more events (upper curve) or less events (lower curve) is <1%. Plots for full length sequences with a single error for the 5C.C7, OT-1, and DO11.10 TCR (A-C), and corresponding plots for sequences with multiple errors (D-F) are shown.

The percent of correct sequence reads in the forward orientation for each TCR showed moderate variability, 52.0%, 46.3%, and 37.5% for the 5C.C7, OT-1, and DO11.10 TCR with a q = 0, and 53.5%, 45.3%, and 39.6% with a q = 30. If errors are introduced in a directionally neutral manner, erroneous sequences should show similar ratios. Phred-unfiltered sequences were segregated by lane due to the prominent lane effect observed (Figure 6) and sequences identified at least 20 times assessed. Sequences incorporating a single nt substitution showed significant directional skewing from the true sequence (Figure 7a-c). For the 5C.C7, OT-1, and DO11.10 TCR, only 14.7%, 18.3%, and 20.2% of sequences with single mutations fell within curves defining a >98% confidence boundary for anticipated sequence direction based on a binomial model. A strong degree of skewing was even seen with sequences for which 100s or 1000s of independent reads were acquired. Skewing was even more dramatic when sequences with multiple errors were assessed (Figure 7d-f). Only 0.81%, 2.8%, and 12.7% of sequences for the 3 TCR respectively fell within the confines of the same binomial boundaries. Therefore, the insertion of errors during high throughput sequencing is highly dependent on the direction of the sequence read.

Application of a phred filter (q = 30) substantially mitigated, though did not wholly eliminate this skewing. Among the erroneous sequences, 48.1%, 70.4%, and 61.9% fell within the binomial boundaries for the 3 TCR (see Additional file 3, Supp. Figure S3a-c). Therefore erroneous sequences show marked directional skewing from that anticipated from the correct sequence, and the extent of this skewing is a function of read quality.

### Nucleotide error propensity

We next analyzed whether error rates varied with specific nt substitutions. As complementary mutations must be inserted during the sequencing step for forward and reverse reads to acquire the same sequence change, we separately assessed forward and reverse nt substitution rates.

**Figure 8:**
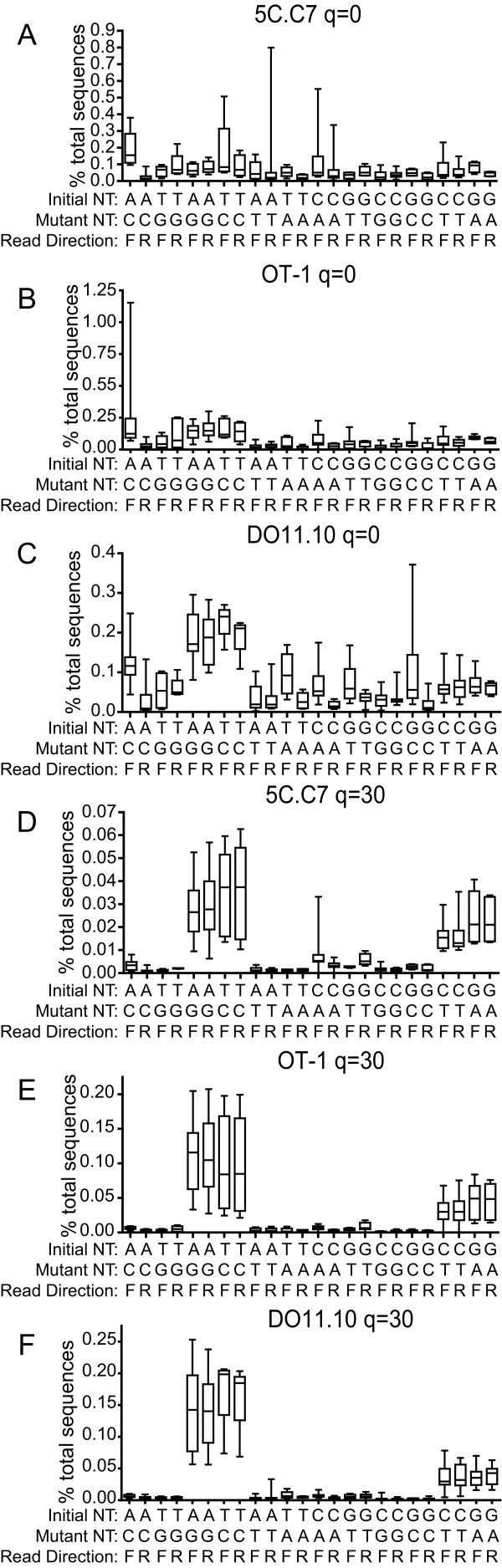
**Rates of specific nt substitutions**. Rates of the indicated nt substitutions at individual positions were tabulated separately for forward and reverse direction reads. The average rate of a given substitution per position bearing the indicated initial nt within 5C.C7 (A, D), OT-1 (B, E), and DO11.10 (C, F) sequence sets was calculated. The median (line), 25-75 percentile error rate (box), and range (whiskers) of this for the 27 sequencing reactions per TCR are plotted for phred unfiltered (A-C) or q = 30 (D-F) filtered sequences.

Among sequences not filtered based on phred score, substantial variability was observed in the rate of specific substitutions. In addition to the variability between specific nt locations within a CDR3 sequence (Figure 6), variability in error rates between different nt substitutions, between substitutions and their complements (e.g. G→A versus C→T), based on sequencing direction, and between the different TCR were evident (Figure 8a-c). This indicates that specific nt substitutions did not prominently influence overall error rates. After filtering at a q = 30, however, clear propensities for specific nt substitutions became apparent (Figure 8d-f). Specifically, errors were primarily accounted for by just 4 of the 12 possible nt substitutions in all of the TCR. Furthermore, these formed 2 pairs of complementary nt substitutions; C→T and G→A, and A→G and T→C. Each of the complementary pairs were observed at similar rates in each of the TCR. Moreover, similar rates of incorporation of these nt substitutions were observed in sequences read in either the forward or reverse directions. Therefore specific nt substitutions have a propensity to occur during high throughput sequencing and this becomes apparent only in sequences with the highest quality scores. The overall rates of these substitutions show symmetry in both sequence direction and between complementary nt changes.

### Assessment of probable erroneous sequences in intact repertoires

**Figure 9:**
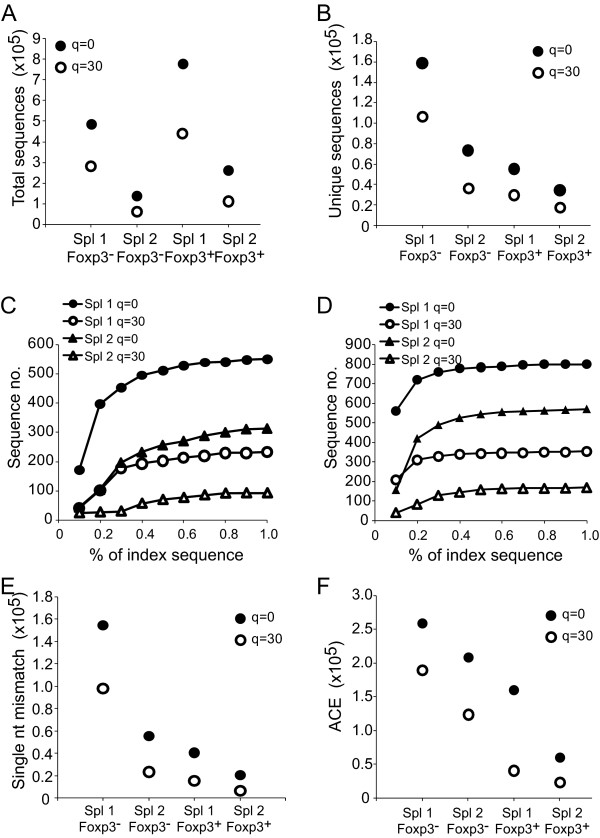
**Analysis of polyclonal C57BL/6 repertoires**. In 2 independent analyses, C57BL/6 splenocytes were sorted into CD4^+^GFP-Foxp3^- ^and CD4^+^GFP-Foxp3^+ ^populations and the Vβ8.2 TCR repertoire analyzed. Frequency of total (A) and unique (B) sequences acquired for each analysis without or with filtering sequences at q = 30. For each unique sequence acquired, sequences present at lower frequency with a single nt mismatch were tabulated. For the 20 most frequent sequences in each cohort, the total number of single nt mismatch sequences present at less than the indicated frequency (abscissa) relative to each corresponding high frequency index sequence were tallied. The total number of these presumed erroneous sequences for the Foxp3^- ^(C) and Foxp3^+ ^(D) populations either analyzed without filtering or filtered at a q = 30 are plotted (ordinate). Results demonstrate a decreased number of presumed erroneous sequences after applying a q = 30 filter. (E) For each unique sequence, the total number of other unique sequences present at a lower frequency and with a single nt mismatch was tallied. The number of these single mismatch sequences was summed for all sequences within each cohort with or without q = 30 filtering. (F) ACE values were calculated as estimates of total repertoire diversity in populations either with or without q = 30 filtering.

To examine the impact of sequence filtering on intact repertoires, we assessed TRBV13-2^+ ^sequences of 2 samples each of flow cytometrically purified CD4^+^Foxp3^+ ^or CD4^+^Foxp3^- ^T cell TCR from C57BL/6 mice. Total sequence numbers varied from 136,716 to 779,107 and unique sequences from 34,449 to 158,886 in the different analyses in the absence of phred-based filtering (Figure 9a, b). Application of a q = 30 filter reduced the total sequence numbers by 49.4 ± 7.7% and unique sequence numbers by 45.0 ± 8.1%.

We next assessed whether application of a q = 30 filter decreased the number of low frequency sequences with single nt mismatches compared with high frequency sequences. To test this, we compared all unique sequences with each other to identify for each sequence all other sequences that had single nt mismatches and were present at a lower frequency. We then used cutoff values of 0.1 - 1% the frequency of the index sequence to indicate probable mis-sequencing events. Numbers of low frequency single nt mismatch sequences for the 20 most frequent sequences in each sequencing cohort were tallied for each cutoff value. This demonstrated that for any single cutoff value, filtering at q = 30 on average reduced the number of lower frequency single nt mismatched sequences by 63 - 71% (Figure 9c, d). As an alternative approach, we also tallied for each sequence in a cohort the total number of other sequences that contained a single nt mismatch and were present at a lower frequency. Application of a q = 30 filter led to a 55.4 ± 13.1% decrease in the total numbers of these sequences in the 4 different cohorts (Figure 9e). Therefore, phred-based filtering effectively reduces the number of likely erroneous sequences.

In order to further assess the impact of increasing the phred cutoff on repertoire composition, we determined the abundance coverage estimator (ACE), a commonly applied indicator of repertoire diversity. ACE values were markedly altered by applying a phred score filter, with a decrease in estimated sequence diversity of 26.9% and 40.9% for the Foxp3^- ^populations and 61.5% and 74.7% for the Foxp3^+ ^populations (Figure 9f). These results illustrate how alterations in sequence fidelity can impact estimates of TCR repertoire size. They further emphasize the caution needed in interpreting sequencing results, as the incorporation of mis-sequence events in repertoire data sets can lead to marked shifts in calculated repertoire diversity.

## Discussion

The profile of errors that accumulate in TCR repertoire data acquired by next generation sequencing has not been fully explored. Our results, derived by sequencing monoclonal TCR CDR3 chains, indicate that an error rate >1% and as high as 6% can be expected after Illumina^® ^sequencing of a 30-36 nt CDR3 central to a 125 bp read, even after screening of sequences for identity with established TCR sequence 5' and 3' of the CDR3. Considering that millions of sequences may be acquired in a single repertoire assessment, tens of thousands of erroneous sequences may be present as well as thousands of unique erroneous sequences. Indeed, if a mean of >100 events are acquired for "correct" unique CDR3 sequence reads, at an error rate of 1% more unique erroneous sequences may be present than error-free sequences. Although our analyses focused on the Illumina^® ^system, the level of error we observed is of a similar magnitude to the ~2% estimated using the Roche platform for shorter (14 nt average) CDR3 region reads [17].

Our results have a number of implications both in regards to the extent to which sequencing errors may permeate repertoire data and methods through which these can be refined. Erroneous sequences had specific characteristics. The large majority showed length identity with the parental CDR3, and among these most incorporated only single nt substitutions (79.1 ± 10.5%, 88.4 ± 5.7%, 88.2 ± 4.9% for 5C.C7, OT-1, and DO11.10 CDR3 at q = 0, and 99.2 ± 0.1%, 98.9 ± 0.2%, and 98.3 ± 0.2% respectively at q = 30). Therefore elimination of single nt substitutions related to an index sequence of higher frequency has the potential to remove the majority of erroneous sequences (Figure 5). Site and nt-specific substitution rates varied substantially within a CDR3, however, and in our data sets did not exceed a frequency of 0.69% for phred unfiltered sequences and 0.24% for sequences filtered at a q = 30. Identifying single nt mismatch sequences compared with a higher frequency index sequence in repertoire analyses can be performed through straightforward algorithms, and a threshold frequency value can be assigned to flag such sequences as potential mis-sequence events. It would seem prudent to incorporate a separately barcoded monoclonal TCR control in all repertoire studies so as to establish error thresholds based on the quality of individual sequencing reactions.

One limitation in filtering single nt mismatch sequences is that for sequences present at low frequencies, there may be insufficient event numbers to reliably identify probable mis-sequence events. For example, if 200 index sequences are present and the filter cutoff is set at 0.5% of the index, a one nt mis-match sequence observed once would be culled (200 × 0.5% = 1). But if that sequence was present twice, it would not meet criteria for this filter. Yet, at these small discrete numbers, a binomial distribution of detected events when the occurrence probability is 0.5% indicates that any potential mis-sequence event present 3 or fewer times would need to be culled to be >95% confident that all sequences less frequent than 0.5% of the index are eliminated. Therefore it may be necessary to establish variable cutoffs as sequence counts become low, so as to reliably eliminate mis-sequences. Increasing cutoff values, however, may also be associated with an increased risk of improperly excluding genuine sequences from the data set. A more complex algorithm that explores sequence space by clustering similar sequences with up to 3 nt substitutions has also been used previously, and our results would support this type of approach [15]. However, that algorithm assumed that genuine CDR3 sequences would only rarely possess <3 nt mismatches. Eliminating sequences with multiple differences may lead to overculling, and if phred filters are applied may be unnecessary. Again, the addition of quantitatively defined frequency thresholds based on internal controls may minimize the inappropriate extraction of true sequences with high nt similarity to an index sequence.

No evidence was found for substantial error introduction during sample preparation steps. Although this phase of manipulation incorporates 2 rounds of PCR, these were performed with high fidelity polymerases with low intrinsic error rates. Errors introduced through PCR would be anticipated to be idiosyncratic. For each of the TCR, among the multiple independently prepared samples, little variation in error rates was observed. As it would seem improbable that the same rare events are consistently incorporated in these steps, the alternative conclusion that the preparatory amplifications did not markedly contribute to overall error seems justified. An alternative source for errors may be those inherent in mRNA transcription within the T cell. However, this would also seem unlikely to be a primary contributor as measured rates of mRNA transcriptional error are 1 - 2 orders of magnitude lower than that observed in these studies [25,26]. This would suggest that the solid phase amplification and sequencing steps are the primary sources of errors, and improved technologies that minimize these are therefore critical. Indeed, unlike single-strand sequencing, more recently developed paired-end approaches that bi-directionally cover the CDR3 sequence may allow improved identification and filtering of sequences with errors.

Our data indicate that additional features may aid in identifying erroneous sequences within mixed TCR populations. Skewed sequencing direction was highly associated with errors. Among phred-unfiltered sequences, the majority of erroneous sequences were observed to be sequenced primarily in a forward or reverse orientation. Indeed, for many sequences, exclusive directionality was observed despite the presence of hundreds of independent reads. Restricting sequences based on phred scores greatly diminished, though did not eliminate this directional bias. Hence, filtering sequences based on directional bias may be useful to eliminate erroneous sequences. One caveat is that in the setting of a large polyclonal population of T cells, even with the acquisition of millions of sequences, many erroneous sequences will be present among lower frequency events. Event numbers may often be inadequate to reliably screen for variation from an anticipated distribution of forward to reverse reads.

Interestingly, with the phred-filtered sequences, a clear nt bias was observed among errors for each of the TCR. Specifically, four substitutions comprised the majority of errors, C→T, G→A, T→C, and A→G. Why these particular purine-pyrimidine transversions are specifically prominent is not clear. However, 2 forms of symmetry underlie them. First, bidirectional mutations are seen, i.e. a C→T and T→C, and a G→A and A→G. This suggests that these base pair combinations are specifically prone to errors. Second, the mutations are also related by their equivalence across complementary read strands. Thus a C→T substitution in a CDR3 sequence could result from a C→T error in a forward read or a G→A in the reverse read. A similar symmetry applies to the T→C and A→G substitutions. This indicates that very specific base pairing is highly prone to error during sequencing. The nature of the base substitution is therefore a potentially important parameter in determining the likelihood that related sequences result from mis-sequencing.

Considering the different factors that are associated with erroneous TCR CDR3 sequences, we would envision a multi-step process for filtering sequences. Most important is the initial application of a phred filter. We observed a progressive decrease in overall errors by increasing the base-call value. Often a q = 20 is considered an adequate cutoff, however our findings indicate a decrease in errors with a q = 30, with an acceptable loss of total sequence numbers. Using single end sequencing here, many residual erroneous sequences were nevertheless present. Paired end sequencing may provide additional benefit in excluding mis-sequence events when full length bidirectional sequence can be acquired. Additional error reduction strategies will depend on the residual error rate present, which can be indicated by a separately barcoded monoclonal CDR3 control incorporated into a sequencing reaction. Our data indicates that at a q = 30, filtering single nt mismatch sequences that are less frequent than 1% of a high frequency index sequence reliably purges >98% of residual erroneous sequences. Increasing the threshold value for filtering potentially incorrect sequences may eliminate more erroneous events but will also increasingly purge true sequences. Further, as mentioned above, as the frequency of the index sequence diminishes to low numbers, the threshold value for culling sequences may need to be increased based on binomial probabilities. It may therefore be helpful to examine these events for additional indications of error, such as the presence of specific transversions that were particularly common among erroneous sequences. Finally, in performing multiple repetitions of sequencing reactions, we observed that many low frequency sequences were only present in one or a few sequencing reactions. The performance of multiple independent sequencing reactions on single amplified samples permits the identification of common sequences, providing increased confidence in their authenticity.

As each sample analyzed for CDR repertoire possesses variable intrinsic diversity, it is not possible to a priori define the extent to which specific interventions that exclude sequences from a data set will mistakenly purge true sequences. Ultimately the best means to safeguard the integrity of CDR3 data set is by diminishing the errors inherent in sequencing, and this must be a priority. New third generation sequencing instruments are currently under development, and, not requiring sample amplification, have the potential to eliminate errors introduced during sample preparation [27]. Fidelity of these systems for quantitative repertoire assessments, however, remains to be determined. This would have to be high as sequencing primary rather than amplified DNA or cDNA samples markedly reduces sequence redundancy, which may be used for quality control.

## Conclusions

In summary, we demonstrate a significant rate of errors introduced during high throughput single-end CDR3 sequencing, with >1% erroneous sequences even after application of quality filters for sequence base call in the CDR3 and for sequence fidelity surrounding it. These errors show high positional and nt-dependent variability. Our results further indicate potential utility in filtering sequences based on single nt mismatches compared with more frequent index sequences, directional skewing of reads, and specific nt substitutions prone to errors. Application of lane-specific monoclonal sequence controls prepared in parallel with experimental samples may aid repertoire analyses by probing the rates of sequence errors within specific lanes and thereby informing algorithms used to cull potentially erroneous sequences.

## Methods

### Mice

D011.10/Rag2, TCR-Cyt-5C.C7-I/Rag2, and OT-1/Rag1 mice were purchased from Taconic Farms, Inc. (Germantown, NY). C57BL/6 GFP-Foxp3 mice were obtained from Dr. A. Rudensky (MSKCC). Experiments were performed in accordance with institutional animal care and use committee guidelines.

### Cell isolation, RNA isolation, cDNA transcription, and amplification

5 × 10^6 ^peripheral lymphocytes were lysed, and total RNA was isolated using RNeasy (Qiagen, Valencia, CA). cDNA was produced using Omniscript RT (Qiagen) per the manufacturer's instructions. cDNA containing each TCR was amplified in triplicate with Cβ (5'-GGGTGGAGTCACATTTCTCAGATC-3') and Vβ3 (5'-GCAGGAGACTCAGCACTGTACCTCT-3'), Vβ5.1 (5'-AGCTAGAGGACTCTGCCGTGTACTTCT-3'), Vβ8.1 (5'-GCTTCCCTTTCTCAGACAGCTGTATATTTC-3') or Vβ8.2 (5'-CCCCCTCTCAGACATCAGTGTAC-3') specific primers for the 5C.C7, OT-1, D011.10, and polyclonal TCR respectively. High Fidelity PCR System (Roche, Indianapolis, IN) was used to amplify CDR3 cDNA with the following PCR conditions: 11 cycles at 95°C 30 sec, 60°C 30 sec, and 72°C 30 sec, followed by 21 cycles at 95°C 30 sec, 50°C 30 sec, and 72°C 20 sec. PCR products were purified by agarose gel electrophoresis and column purification (QIAquick Gel Extraction Kit, Qiagen).

### DNA preparation and sequencing

DNA end repair was performed by incubating the purified PCR products with 15 U T4 DNA Polymerase (NEB, Beverly, MA), 50 U T4 Polynucleotide Kinase (NEB), 0.4 mM 2'-deoxynucleoside 5'-triphosphate, T4 ligase buffer with 10 mM 2'-deoxyadenosine triphosphate (Promega, Madison, WI), and 5 U Klenow enzyme (Promega) for 30 min at 20°C. The products were purified using the QIAquick PCR Purification Kit (Qiagen). To adenosine tag the DNA 3' ends, purified DNA was incubated with 25 U Klenow fragment (3' - 5' exo minus; NEB), Klenow buffer, and 0.2 mM 2'-deoxyadenosine triphosphate for 30 min at 37°C. The product was purified and concentrated to 10 μl using the MinElute Reaction Cleanup Kit (Qiagen). Next, sequencing adapters were ligated onto the PCR products, using 3 mM Index PE adapter Oligo Mix, 5 μl Quick DNA ligase (NEB), and ligase buffer, and incubated for 15 min at 20°C. To remove unligated adapters, the product was purified using the QIAquick gel purification kit (Qiagen). Samples were each divided into 3, and InPE 1.0 and 2.0 (Illumina^®^, San Diego, CA) and Index primers were next linked to the DNA, using the Phusion DNA Polymerase Kit (Finnzymes Oy, Eskoo, Finland) with the following PCR condition: 19 cycles at 98°C 10 sec, 65°C 30 sec, 72°C 30 sec. Additional index primer sequences were manufactured by the St. Jude Hartwell Center. The PCR products were purified using the QIAquick PCR purification kit, as above. Each sample was divided into 3, and equimolar quantities of each sequenced over three lanes of a flow cell with an Illumina^® ^Genome Analyzer IIx sequencer using a 125 bp (plus 6 bp barcode) recipe to obtain single-end reads that cover the entire CDR3β region.

### Data analysis

Raw data was demultiplexed and filtered using CASAVA 1.6.0. The data was subsequently trimmed for the presence of adapter sequences using CLCGenomics WorkBench v4.0. The Illumina^® ^125-bp reads were then scanned for Vβ and Jβ sequence homology immediately external to the C and F residues bordering the CDR3 using cross_match http://www.phrap.org/. 27 nt long segments for each Jβ and 25, 27, and 30 nt segments respectively for Vβ3, Vβ5.1, and Vβ8.1 were mapped to each sequence. To identify CDR3 sequences, reads were filtered based on the cross_match results using the following criteria: (i) 100% sequence identity for both Vβ and Jβ mapping; (ii) translated amino acid sequence between Vβ and Jβ is in the correct frame and reveals a translated product (no stop codon); (iii) the deduced CDR3 amino acid sequences between the Vβ and Jβ sequences begin with the conserved C and end with a FGXG, FAXG or HGXG motif. The deduced CDR3 nt sequences were then scanned using the Phred quality score cutoffs of 0, 10, 20 or 30 [24], and reads with CDR3 nt sequence containing at least one low-quality base at a given cutoff level were filtered out. ACE values (n = 10) were calculated using EstimateS software v8.2.0.

## Authors' contributions

PN was responsible for experimental design and analyses, and assisted in writing the manuscript. JM performed data analyses and result interpretation, and assisted in writing the manuscript. DP and CC performed statistical analyses. CO assisted with experimental design, result interpretation, and writing the manuscript. TG was responsible for project coordination and design, data analysis and writing the manuscript. The manuscript was read and approved by all authors.

## Supplementary Material

Additional file 1 **Supplemental Figure S1**. Selective exclusion of erroneous sequences with increasing phred cutoff. Click here for file

Additional file 2 **Supplemental Figure S2**. Position and nt specific substitutions in phred-filtered data sets. Click here for file

Additional file 3 **Supplemental Figure S3**. Directional skewing of phred-filtered sequences. Click here for file
