# Supplementary material for: Identification of errors introduced during high throughput sequencing of the T cell receptor repertoire
Source: BMC Genomics. 2011 Feb 11;12:106. doi: 10.1186/1471-2164-12-106 (PMC3045962; doi:10.1186/1471-2164-12-106)
Supplement: Additional file 1 — Supplemental Figure S1. Selective exclusion of erroneous sequences with increasing phred cutoff. [file 1471-2164-12-106-S1.PDF]

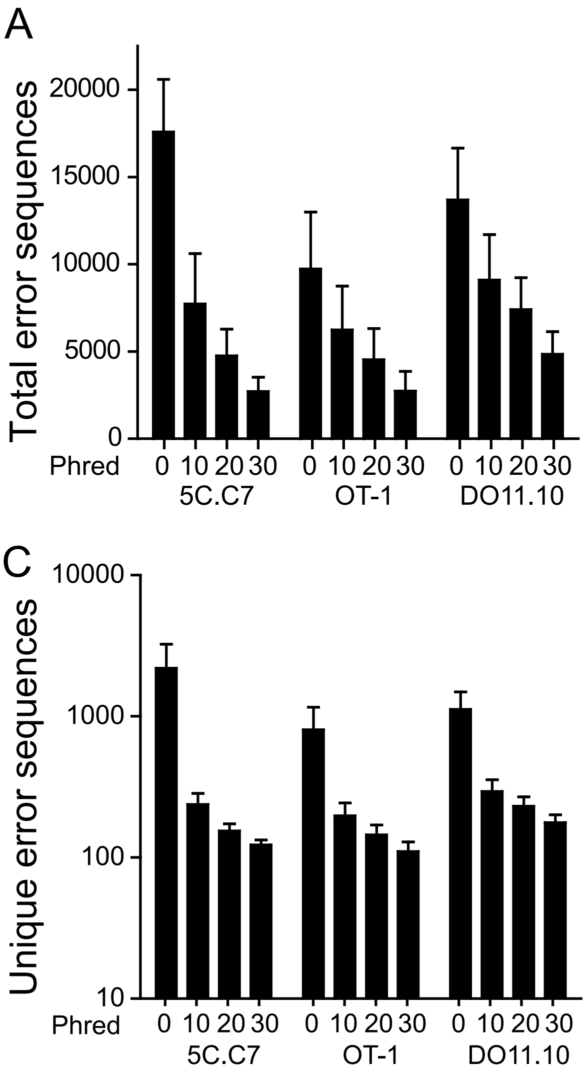

Supplemental Figure S1. Selective exclusion of erroneous sequences with increasing phred cutoff. (A) Total number of erroneous sequences for each TCR sample is plotted as a function of phred score for each of the 3 TCR. (B) Total number of sequences is plotted as in (A). (C) Number of unique erroneous sequences is shown. Mean+1 S.D. is plotted in (A-C) for the 27 samples sequenced per TCR. (D) Data from the independent sequencing reactions were pooled, and number of unique erroneous sequences for aggregated data plotted as a function of phred score.
