# Supplementary material for: Identification of errors introduced during high throughput sequencing of the T cell receptor repertoire
Source: BMC Genomics. 2011 Feb 11;12:106. doi: 10.1186/1471-2164-12-106 (PMC3045962; doi:10.1186/1471-2164-12-106)
Supplement: Additional file 2 — Supplemental Figure S2. Position and nt specific substitutions in phred-filtered data sets. [file 1471-2164-12-106-S2.PDF]

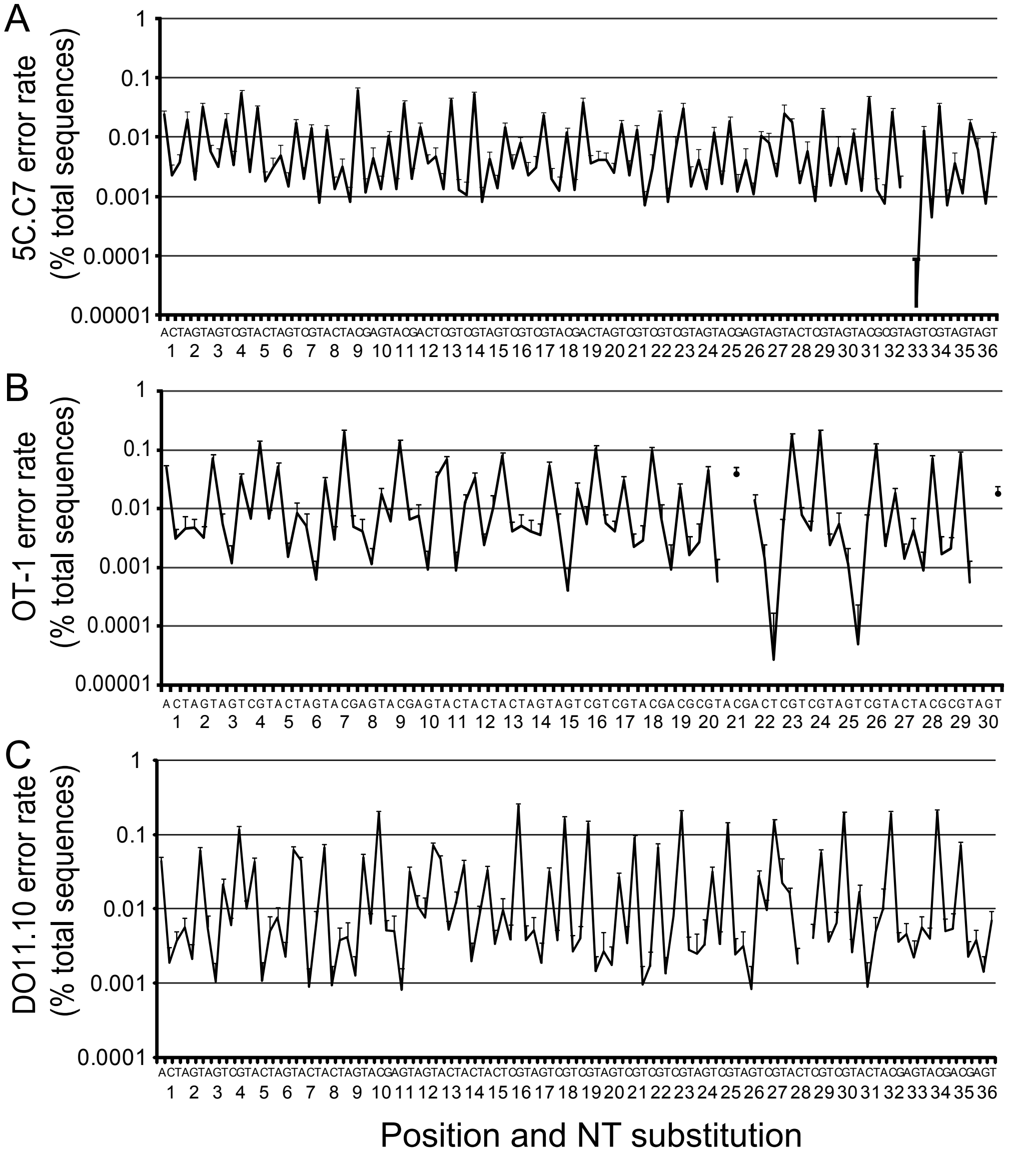

Supplemental Figure S2. Position and nt specific substitutions in phred-filtered data sets. Analyses were performed as in figure 6 for sequences derived from the 5C.C7 (A), OT-1 (B), and DO11.10 (C) TCR except, due to the low variance across lanes, mean+1 s.d. of all 27 samples per TCR is plotted. Positions without identified errors are indicated by gaps in the plots. Plotted lines are shown to aid visualization of results and do not indicate continuity among x-axis variables.
