# Supplementary material for: Identification of errors introduced during high throughput sequencing of the T cell receptor repertoire
Source: BMC Genomics. 2011 Feb 11;12:106. doi: 10.1186/1471-2164-12-106 (PMC3045962; doi:10.1186/1471-2164-12-106)
Supplement: Additional file 3 — Supplemental Figure S3. Directional skewing of phred-filtered sequences. [file 1471-2164-12-106-S3.PDF]

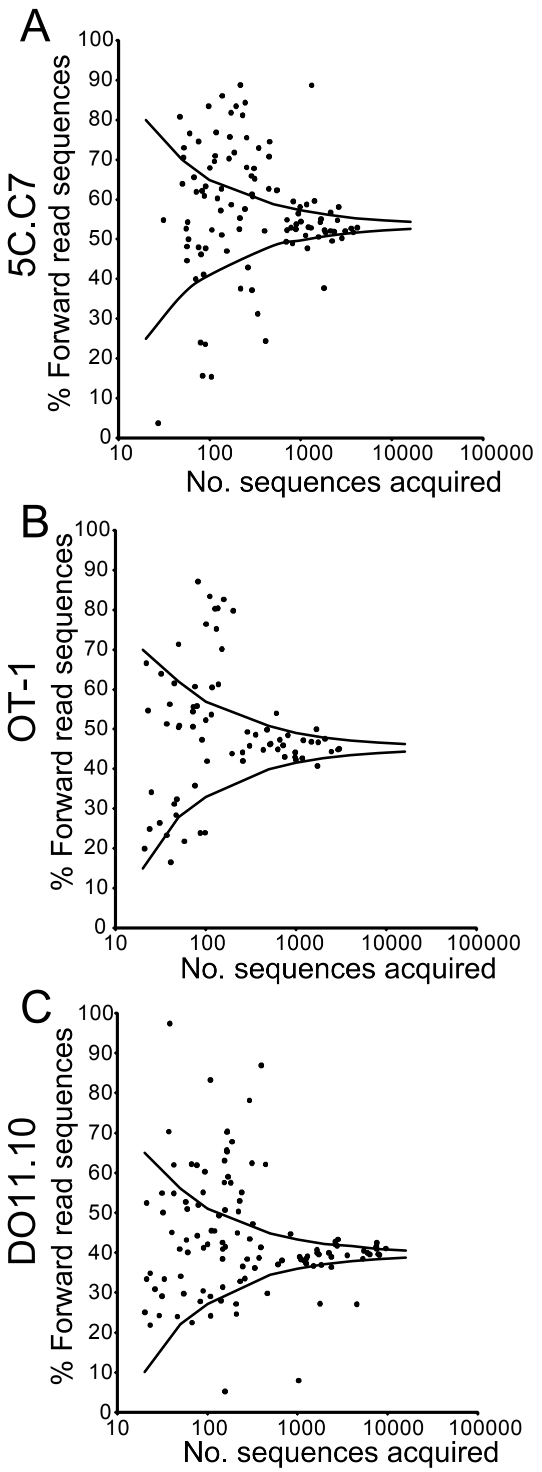


**Supplemental Figure S3. Directional skewing of phred-filtered sequences.** Analyses were performed as in fig. 7 (main text) using data sets of sequences filtered at a q=30, except sequences were pooled across lanes due to the diminished lane-effect on error rates for the filtered sequences. (A-C) Plots for the 5C.C7, OT-1, and DO11.10 TCR are shown. Because of the small number of erroneous sequences identified at a frequency >20 with multiple errors, plots combine sequences regardless of the number of errors they contain.
